# Postablation assessment of hepatocellular carcinoma using dual-energy CT: Comparison of half versus standard iodine contrast medium

**DOI:** 10.1371/journal.pone.0219577

**Published:** 2019-07-09

**Authors:** Yuan-Mao Lin, Yi-You Chiou, Mei-Han Wu, Shan Su Huang, Shu-Huei Shen

**Affiliations:** 1 Department of Radiology, Taipei Veterans General Hospital, Taipei, Taiwan; 2 School of Medicine, National Yang-Ming University, Taipei, Taiwan; 3 Department of Medical Imaging, Cheng Hsin General Hospital, Taipei, Taiwan; Texas A&M University, UNITED STATES

## Abstract

This retrospective study was aimed to evaluate the reduced iodine load on image quality and diagnostic performance in multiphasic hepatic CT using a novel monoenergetic reconstruction algorithm (nMERA) in assessment of local tumor progression after radiofrequency ablation (RFA) of hepatocellular carcinoma (HCC). Ninety patients who underwent CT 1 month after RFA of HCC. Forty-five patients had multiphasic hepatic dual-energy CT with a half-reduced contrast medium (HRCM) of 277.5 mg I/kg. The nMERA (40–70-keV) images were reconstructed in each phase. Another 45 patients received a standard contrast medium (SCM) of 555 mg I/kg, and the images were reconstructed as a simulated 120-kVp images. Primary outcome was accuracy, sensitivity, specificity, and area under the receiver operating characteristic curve (AUC) in assessment of local tumor progression. Additional advanced assessments included the image noise, attenuation value, contrast-to-noise ratio (CNR), and subjective image quality between the groups. The accuracy, sensitivity and specificity of nMERA HRCM images were 95.7%, 100% and 93.9% for 40 keV, 95.7%, 85.7% and 100% for 50 keV, 83.0%, 42.8% and 100% for 60 keV, and 83.0%, 42.9% and 100% for 70 keV. The AUROC was 0.99, 0.99, 0.94, and 0.93 for 40–70 keV nMERA HRCM images, respectively. Compared with simulated 120-kVp SCM images, nMERA HRCM images demonstrated comparable noise at 70-keV (P < 0.05), and comparable CNR at 40- and 50-keV (P < 0.05). nMERA DECT enables the contrast medium to be reduced to up to 50% in multiphasic hepatic CT while preserving diagnostic accuracy.

## Introduction

Multidetector computed tomography (CT) is widely applied clinically. However, the concerns of radiation exposure, contrast medium-induced nephropathy (CIN), and the dose of contrast medium (CM) have been raised considering that CT is a commonly used tool for disease follow-up [1–3]. Achieving adequate contrast enhancement while using the least amount of CM and ensuring the lowest radiation exposure is the goal of contrast-enhanced CT examination [2]. Therefore, many studies have adopted strategies to reduce the CM dose [4–10].

There are many CT techniques, such as single-energy CT with low tube voltage and dual-energy CT (DECT) with virtual monochromatic images (VMI) or nonlinear blended images, have been reported as CM-dose-reduction strategies for abdominal CT [4–6, 8, 9, 11, 12]. The target organs are liver, kidney, pancreas, arteries, etc, where contrast enhancement difference is diagnostically important in tumor assessment. The quality of images obtained at the low tube voltage of single-energy CT with an iterative reconstruction algorithm and at a reduced CM dose was not inferior compared with that of conventional 120-kVp images [4, 7, 8, 12, 13]. As the tube voltage decreases, the mean photon energy is closer to the k-edge of iodine, resulting in an increase in iodine attenuation. However, the image noise increases as the number of transmitted photons decrease. The iterative reconstruction algorithm was introduced to overcome the drawback, and this algorithm has been shown to improve image quality [4, 12]. However, single-energy CT image reconstruction using this algorithm cannot provide more information than DECT with VMI at an arbitrary energy level. Furthermore, obtaining VMI at a selected energy level can optimize contrast, and this strategy is promising for reducing the CM dose in different CT technologies [5, 9–11]. Several studies have demonstrated various advantages of DECT for imaging and assessment of patients with vascular and oncologic lesions [14–17]. In DECT, a novel monoenergetic reconstruction algorithm (nMERA) was introduced to optimize image quality at low keV levels, providing markedly increased iodine contrast with moderate noise [18–21]. However, this application has not been investigated for CM dose reduction in abdominal CT.

We hypothesize that the CM dose can be reduced without compromising on image quality and diagnostic performance by using nMERA in multiphasic abdominal CT. In this study, we evaluated the application of nMERA and the half-reduced CM dose in assessment of local tumor progression after radiofrequency ablation (RFA) of hepatocellular carcinoma (HCC), and we also aimed to determine the optimal energy level.

## Materials and methods

### Patients

This retrospective study was conducted with the approval of the Taipei Veterans General Hospital Institutional Review Board, and written informed consent was waived from all the patients. Between June 2017 and April 2018, 90 patients underwent dual energy multiphasic hepatic CT one month after receiving RFA of HCC were included in this study. Among these 90 patients, no one met the following exclusion criteria: age less than 18 years and the body mass index (BMI) greater than 32 kg/m^2^. Forty-five patients received half-reduced CM dose (HRCM) because of serum creatinine levels greater than 2.0 mg/d, and others received standard contrast medium (SCM) dose. Patient characteristics are summarized in Table 1.

**Table 1:** Patient characteristics of each groups.

	Standard contrast medium group (n = 45)	Half reduced contrast medium group (n = 45)	P value
All patients	45	45	
Male	36 (80%)	31 (68.8%)	0.33^a^
Age (years)	68.4 ± 1.5	70.6 ± 1.9	0.36^b^
Body mass index (kg/m^2^)	24.7 ± 0.4	25.1 ± 0.6	0.64^b^
Iodine load (g)	35.9 ± 5.8	19.1 ± 4.0	<0.01^b^
CTDIvol (mGy)	27.1 ± 1.0	26.1 ± 0.7	0.42^b^
DLP (mGycm)	891.9 ± 41.2	840 ± 29.8	0.31^b^
Patients with local tumor progression	9 (20%)	12 (26.7%)	0.61^a^
Male	8 (88.8%)	7 (58.3%)	0.65^a^
Age (years)	65 ± 10.2	68.6 ± 9.8	0.54^c^
Body mass index (kg/m^2^)	23.5 ± 2.9	23.1 ± 3.4	0.86^c^
Iodine load (g)	35.3 ± 4.8	17.5 ± 3.4	<0.01^c^
Tumor size (cm)	2.3 ± 1.1	1.3 ± 0.3	<0.01^c^

^a^ Fisher’s exact test,

^b^ Student’s t test,

^c^ Mann-Whitney test

### Imaging protocol of CT

For all patients, CT scans were performed on a second-generation DECT system (Definition Flash, Siemens Medical Solutions, Forchheim, Germany) in the supine position. Unenhanced and two-phase contrast-enhanced images were acquired in hepatic arterial and portal venous phases (HAP and PVP, respectively). HAP and PVP scans were performed in the dual-energy mode (Tube A = Sn140 kVp; tube B = 100 kVp), and the detector configuration was 32 mm x 0.6 mm; the rotation time was 0.5 s; and the helical pitch was 0.6. Automatic current modulation for exposure control (Care Dose 4D; Siemens Healthcare) with quality reference amperages of 178 and 230 mAs for tube A and tube B, respectively, was used.

The iodine contrast agent (Iopamiro 370, Bracco spa, Milano, Italy) at dose of 1.5 mL/kg was delivered to the SCM and HRCM groups with iodine dose of 555 mg I/kg and 277.6 mg I/kg, respectively. CM was administered to all patients by using a dual-chamber mechanical power injector (Stellant CT Injection System, Medrad, Inianola, USA) at a rate of 2.5–3 mL/s, followed by 30 mL of saline chaser through an IV catheter inserted into the antecubital vein.

Intravenous contrast injection technique was based on a bolus tracking method with a threshold of 150 HU from a region of interest (ROI) placed in the descending aorta at the level of the diaphragm. HAP and PVP were started with a total delay of 15 and 60 seconds after reaching the trigger threshold. Dual-energy images were reconstructed on a workstation equipped with dedicated software (syngo.via, version VB10B, Siemens Healthcare); moreover, images were reconstructed with a section thickness of 5 mm and an increment of 5 mm by using a soft tissue convolution kernel (Q30f) and an iterative reconstruction technique (SAFIRE, Siemens; strength level: 3). In the SCM group, images were reconstructed using a linear blended method with a ratio of 0.5, simulating 120-kVp images. In the HRCM group, the images were reconstructed with nMERA at 40-, 50-, 60-, and 70-keV levels.

### Quantitative analysis

A radiologist with 4 years of experience in abdominal CT performed quantitative measurements on the workstation but did not participate in subsequent qualitative image interpretation. Attenuation measurements were obtained manually by placing round or oval ROIs within the aorta, liver, and psoas muscle in the HAP, and within the liver, portal vein, and psoas muscle in the PVP. Two measurements (approximately 4 cm^2^) were obtained within the right and left lobes of the liver at the level of the hepatic hilum, avoiding the great vessels and the focal lesion. Average values were used for analysis. One measurement (approximately 1 cm^2^) was obtained within the lumen of the main portal vein, and one measurement (approximately 2 cm^2^) was obtained within the lumen of the aorta at the level of the main portal vein, avoiding the wall calcification. Finally, two measurements (approximately 2 cm^2^) were obtained within each side of the psoas muscle at the L2 vertebral level, and average values were used for analysis. All attenuation measurements were performed twice to ensure the consistency and reproducibility of results. The size and location of the ROIs were kept consistent in all measurements of each phase.

We defined image noise as the standard deviation (SD) of attenuation measurements within the psoas muscle. The contrast-to-noise ratio (CNR) of the liver and aorta was calculated using the following formula:

$$CNR=\frac{{\text{HU}}_{T}-{\text{HU}}_{muscle}}{Image\;noise}$$

where HU_*T*_ is the mean attenuation of the tissue (liver and aorta), and HU_*muscle*_ is the mean attenuation of the psoas muscle.

The CNR of the portal vein in the PVP was calculated using the following formula:

$$CNR=\frac{{\text{HU}}_{portal\;vein}-{\text{HU}}_{liver}}{{\text{SD}}_{liver}}$$

where the HU_*portal vein*_ is the mean attenuation of the portal vein, HU_*liver*_ is the mean attenuation of the liver, and SD_*liver*_ is the SD of the liver.

### Qualitative analysis

Two radiologists with 15 and 20 years of experience, respectively, in abdominal CT independently assessed the quality of the images reconstructed on the workstation; the radiologists were blinded to the acquisition protocols. All randomly ordered images were initially presented on a soft tissue window with a width of 400 HU and a level of 40 HU, but the readers were allowed to change the window settings as necessary. Using a 5-point Likert scale, the radiologists graded image contrast, noise, artifact, and overall image quality as follows: 1, poor; 2, suboptimal; 3, acceptable; 4, good; and 5, excellent.

### Diagnostic performance and lesion analysis

To assess the local tumor progression, the diagnosis was confirmed by the consensus of two study coordinators reviewing the nMERA HRCM and SCM images and the follow up CT or MRI within two months after the RFA. According to the standardized terminology of the Interventional Working Group on Image-Guided Tumor Ablation, the local tumor progression was considered when a newly developed focal enhancing lesion in the ablation zone, or distortion in the smooth interface of the ablation zone or increase size of the zone were observed [22]. The size, number, and location of lesions were documented. Twenty-five lesions in 21 patients were identified in the study population, comprising 11 lesions in 9 patients of the SCM group and 14 lesions in 12 patients of the HRCM group. The RFA and the intraoperative biopsy were performed in all the lesions. The biopsy results demonstrated 21 (84%) lesions (9 in HRCM group, and 12 in SCM group) with diagnosis of HCC, and 4 (16%) with non-diagnostic results.

The diagnostic accuracy was analyzed in nMERA HRCM group by two radiologists with 15 and 20 years of experience, respectively, in abdominal CT without knowledge of tumor existence. The size, number and location of the lesions were recorded, and finally the ratings were provided as follows: 1, definitely absent; 2, probably absent; 3, indeterminate; 4, probably present; and 5, definitely present.

For quantitative analysis of lesions in nMERA HRCM and SCM groups, the attenuation of the tumor and the surrounding liver parenchyma was measured, and the difference in the attenuation was calculated. The CNR of the tumor was calculated by dividing the calculated difference by the SD of the surrounding liver parenchyma. Measurements were taken by one radiologist with 4 years of experience in abdominal CT.

### Statistical analysis

All numeric values are reported as mean ± SD, and categorical variables are expressed as counts and proportions. The normality of distributions was confirmed using the Kolmogorov–Smirnov test. Two-tailed Student’s *t* test or Mann–Whitney test was used to compare age, BMI, iodine load, radiation dose, and tumor size between the two patient groups, and Fisher’s exact test was used to determine differences between categorical variables. The Mann–Whitney U test was used to analyze differences between the SCM and HRCM groups for quantitative analysis of noise, attenuation and CNR and score of qualitative analysis.

The receiver operating characteristic (ROC) analysis of all lesions was performed tumor by tumor [23]. Diagnostic accuracy was assessed by calculating the area under the ROC curve. The sensitivities for detecting HCC were determined by the number of HCC lesions assigned a score of 4 or greater whereas the specificities were determined by the number of non-HCC lesions that were assigned a score of 0 to 3. Sensitivities and specificities were statistically calculated with the McNemar test. For assessing interobserver variability in qualitative image analysis, kappa statistics were used to quantify the degrees of agreement. For all comparisons, statistical significance was set at p < 0.05. All statistical analyses were performed using SPSS software (SPSS, version 16, SPSS Inc., Chicago, IL, USA).

## Results

### Quantitative image assessment

**Fig 1:**
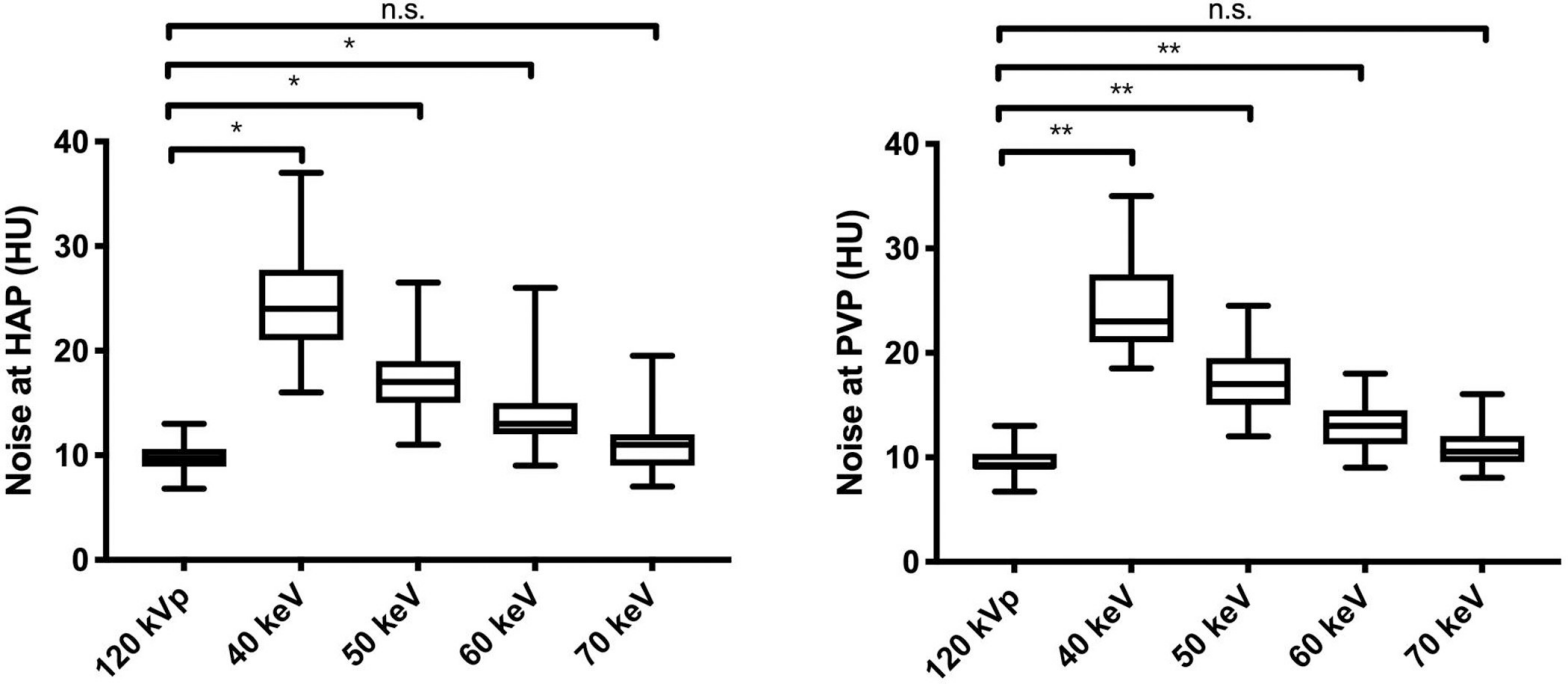
Box-and-whisker plots of image noise of simulated 120-kVp SCM images and 40–70-keV nMERA HRCM images during HAP and PVP. Boxes show the upper and lower quartiles, with median values being shown by the horizontal lines within the boxes. The whiskers represent maximum and minimum values. n.s. p > 0.05, * p < 0.05, ** p < 0.01.

In the HRCM group, the image noise decreased as the voltage increased from 40- to 70-keV in both the HAP and PVP of nMERA images. The lowest noise was found for nMERA images at 70-keV, and there was no significant difference with simulated 120-kVp images in the SCM group (p ≥ 0.05) (Fig 1).

**Fig 2:**
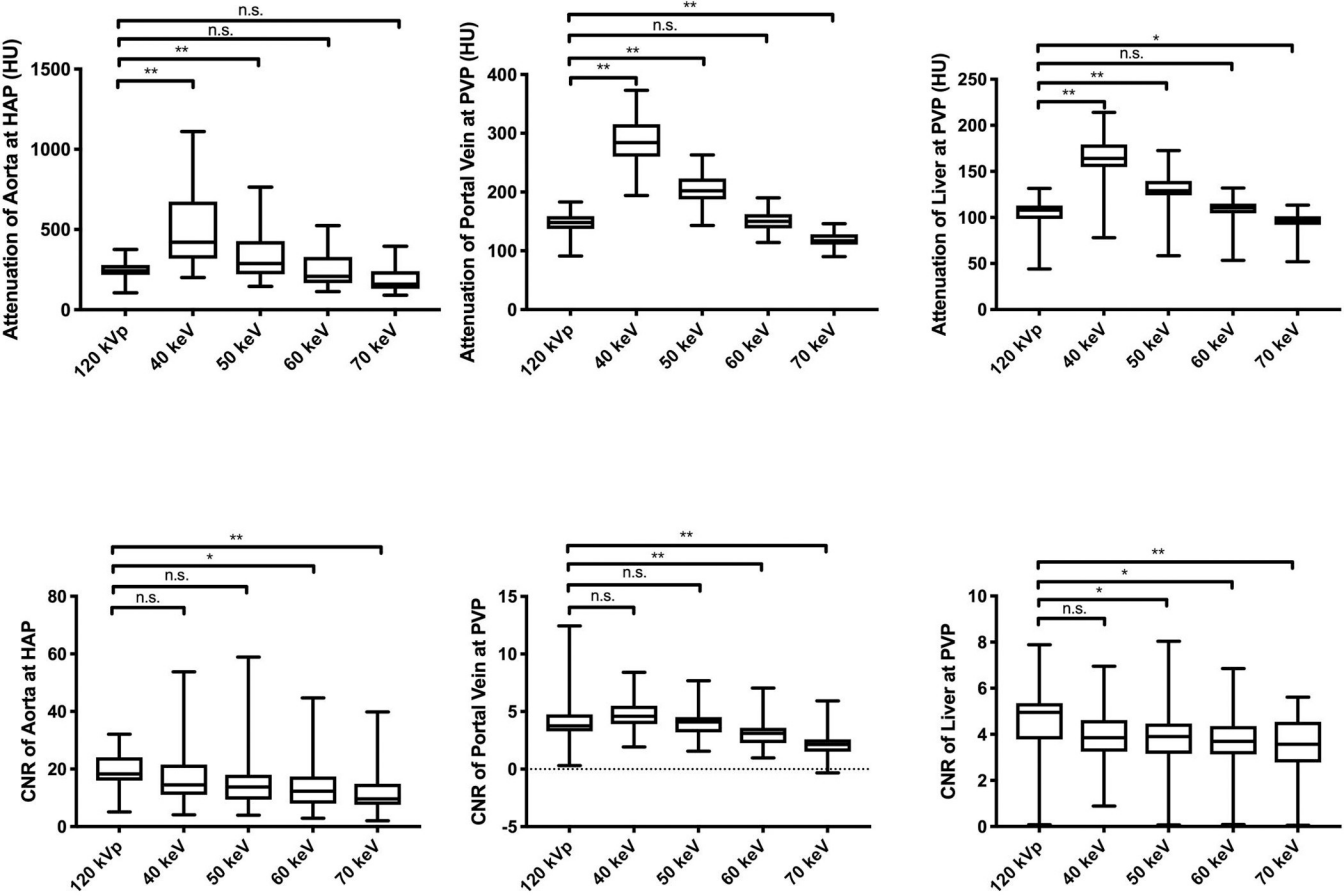
Box-and-whisker plots of the attenuation value and CNR of simulated 120-kVp SCM images and 40–70-keV nMERA HRCM images during HAP and PVP. Boxes show the upper and lower quartiles, with median values being shown by the horizontal lines within the boxes. The whiskers represent maximum and minimum values. n.s. p > 0.05, * p < 0.05, ** p < 0.01.

The aorta, liver, and portal vein attenuation of nMERA images in the HRCM group increased as energy decreased and reached a level equivalent to that of simulated 120-kVp SCM images at 60 keV. Similarly, the CNR gradually increased and energy decreased, and the results revealed no significant difference between the 40-keV nMERA HRCM images and the simulated 120-kVp SCM images (Fig 2).

**Fig 3:**
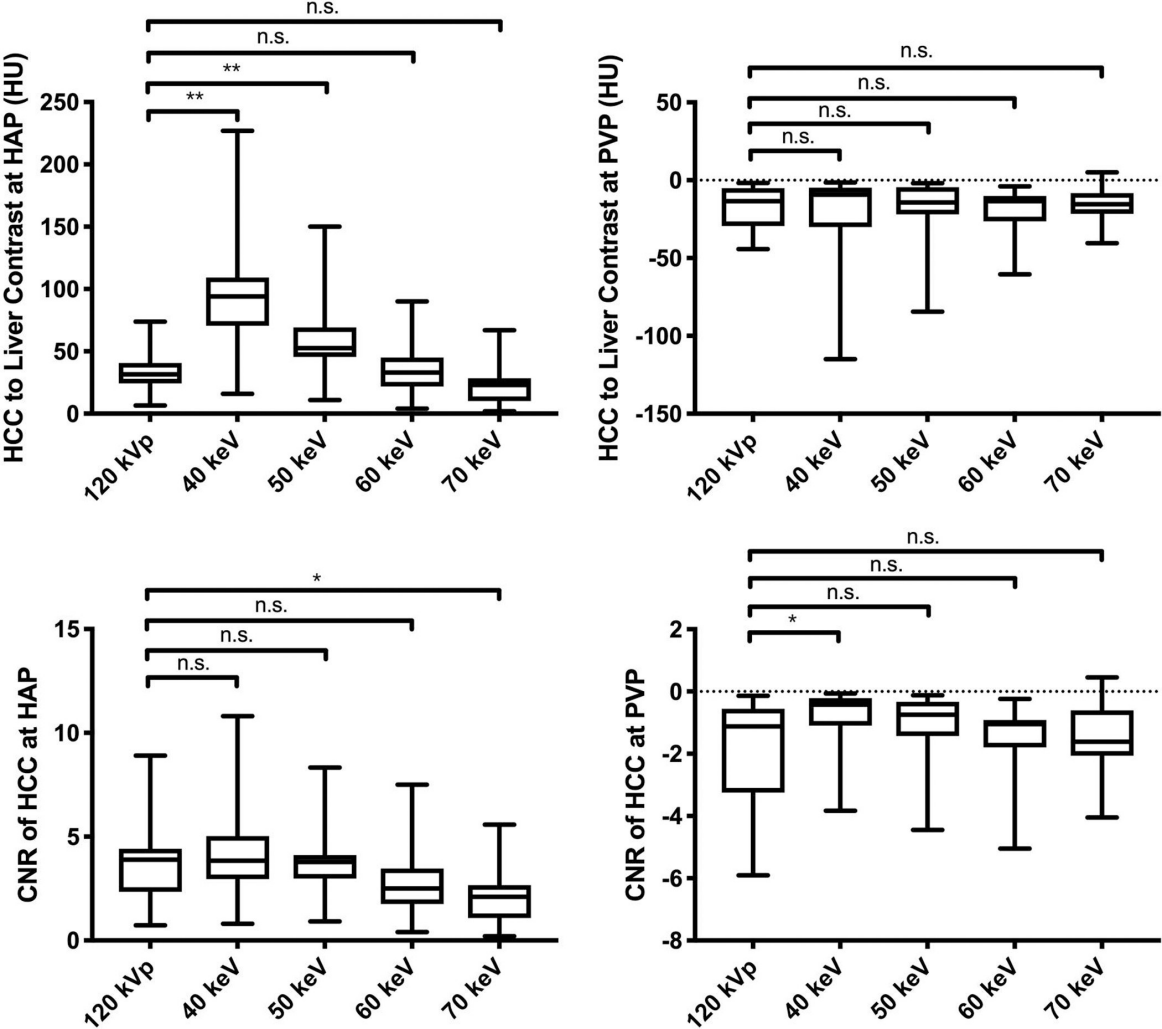
Box-and-whisker plots of the tumor to liver contrast and CNR of tumor in simulated 120-kVp SCM image and 40–70-keV nMERA HRCM images during HAP and PVP. Boxes show the upper and lower quartiles, with median values being shown by the horizontal lines within the boxes. The whiskers represent maximum and minimum values. n.s. p > 0.05, * p < 0.05, ** p < 0.01.

**Fig 4:**
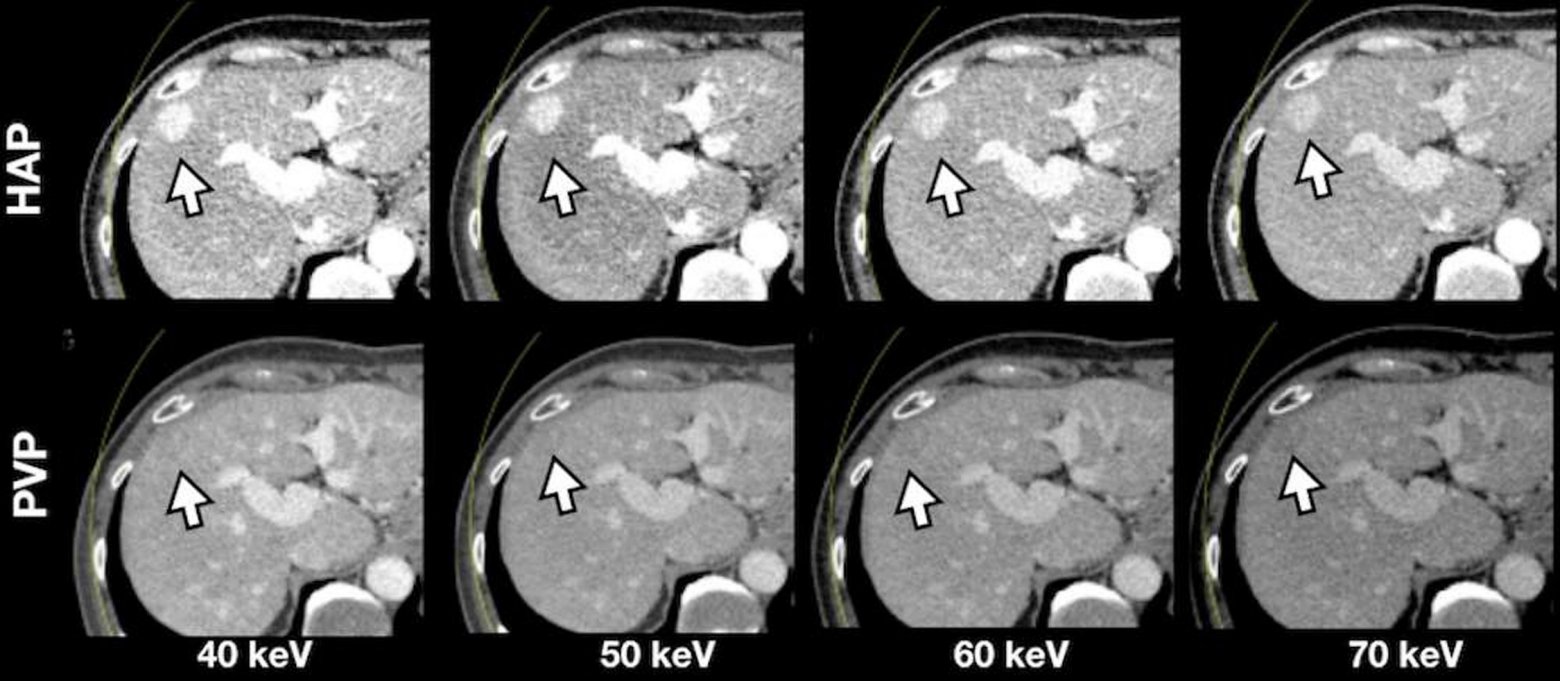
A representative case of a 68-year-old man with local tumor progression. A 68-year-old man (body weight, 70.4 kg; BMI, 24.1 kg/m^2^) underwent multiphasic hepatic CT with dual source DECT at half reduced contrast medium dose. The image was reconstructed with nMERA images at 40–70-keV. All the images were under optimized window width/center setting to each image. The conspicuity of the hypervascular HCC (arrows) gradually improves as the energy level decreases in nMERA images, especially in the HAP image.

The highest tumor-to-liver attenuation contrast was detected in 40-keV nMERA HRCM images, and the contrast decreased as the energy increased (Fig 3). The CNR of the tumor was the highest in 40-keV nMERA HRCM images, and no statistical difference was observed between 40–60-keV nMERA HRCM images and the simulated 120-kVp SCM images (Fig 3). A representative case is shown in Fig 4.

### Qualitative image assessment

The mean score of qualitative assessment is presented in Table 2. The score of image contrast in 40- and 50-keV nMERA HRCM images were equivalent to that of image contrast in the simulated 120-kVp SCM images, and 60- and 70-keV nMERA images had a lower score. The image noise and streak artifact in the SCM images were equivalent to those in 60- and 70-keV nMERA images (p > 0.05) and were superior to those in 40- and 50-keV nMERA images (p < 0.05). The highest score of the image noise and streak artifact was obtained for 70-keV nMERA HRCM images. The overall quality of all nMERA HRCM images were rated lower than the simulated 120-kVp SCM images, and in the HRCM group, 60-keV nMERA images had the highest score.

**Table 2:** Qualitative image analysis.

	Standard contrast medium	Half reduced contrast medium								κ value	P value
	Simulated 120-kVp	40-keV nMERA	P value	50-keV nMERA	P value	60-keV nMERA	P value	70-keV nMERA	P value		
Image Contrast	3.5 ± 0.6	3.5 ± 0.6	> 0.9	3.3 ± 0.7	0.71	2.8 ± 0.8	< 0.001	2.1 ± 0.9	< 0.001	0.521	< 0.001
Image Noise	2.9 ± 0.2	2.3 ± 0.5	< 0.001	2.6 ± 0.6	0.01	3.0 ± 0.6	> 0.9	3.2 ± 0.6	> 0.9	0.932	< 0.001
Streak artefact	3.2 ± 0.4	2.2 ± 0.4	< 0.001	2.7 ± 0.7	0.001	3.4 ± 0.6	> 0.9	3.5 ± 0.6	0.3	0.712	< 0.001
Overall Quality	3.3 ± 0.6	2.6 ± 0.7	< 0.001	2.7 ± 0.8	0.002	2.9 ± 0.8	0.03	2.8 ± 0.7	0.04	0.651	< 0.001

### Diagnostic performance

**Fig 5:**
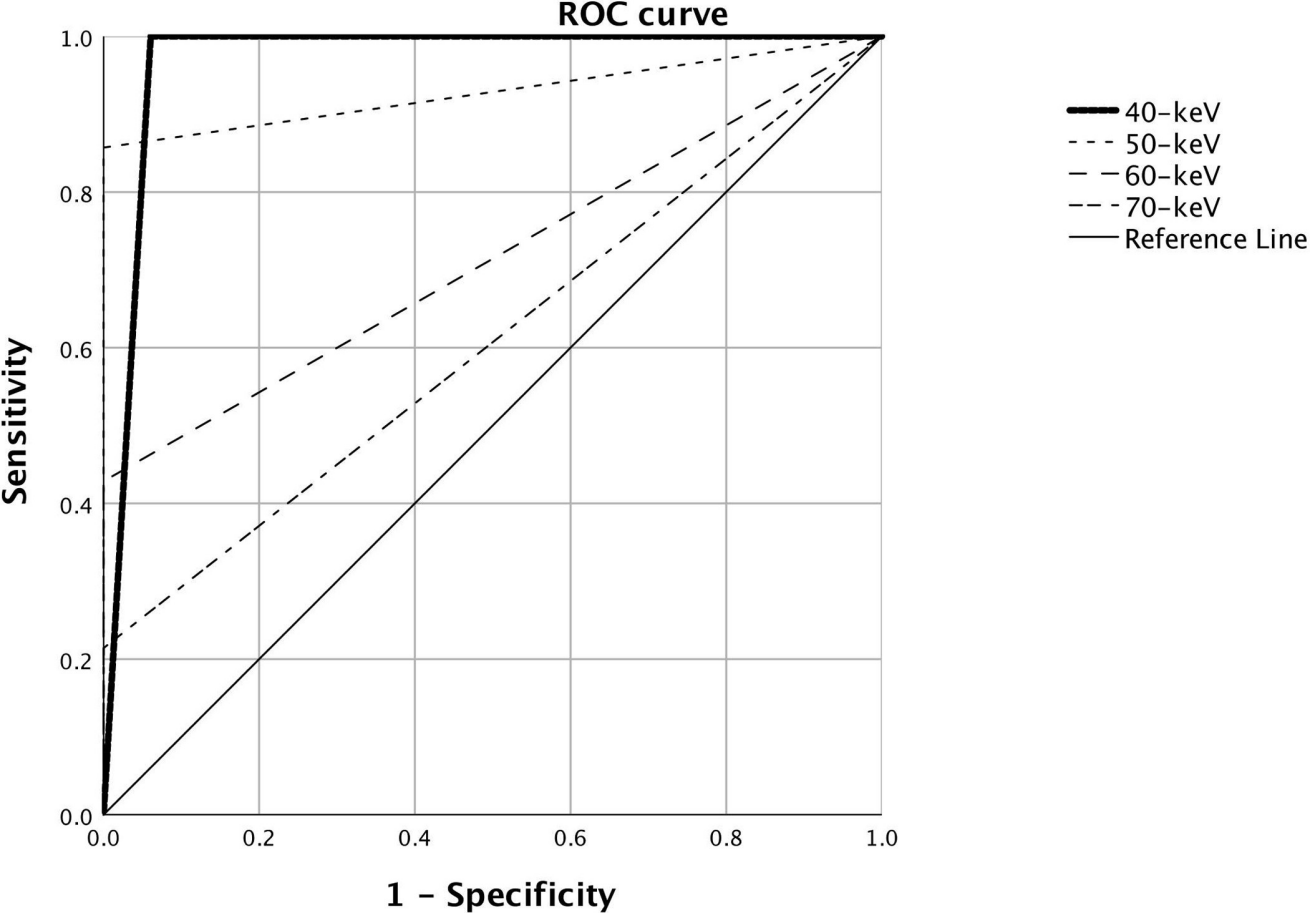
ROC curves of HRCM groups.

**Fig 6:**
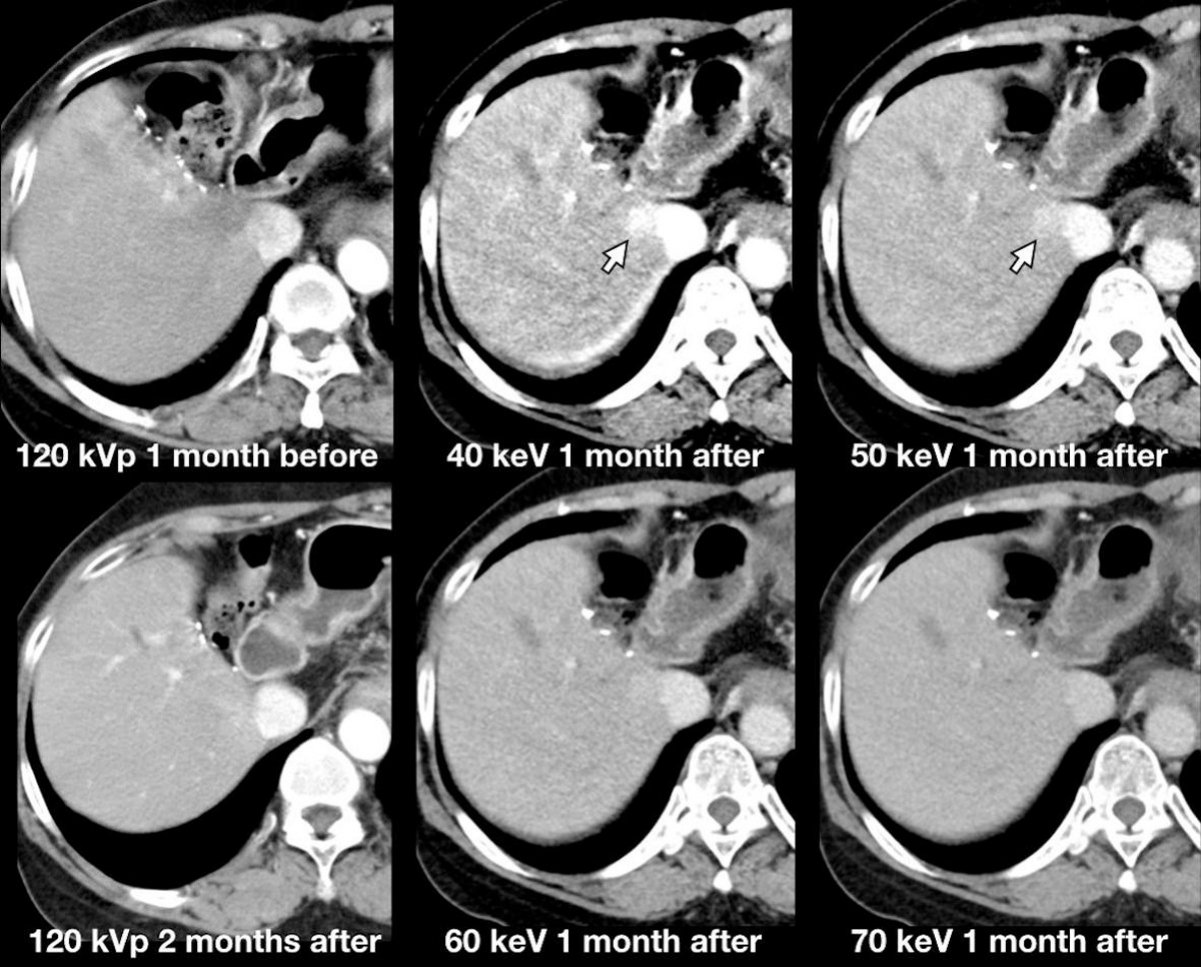
A 64-year-old woman underwent CT for HCC one month before, one month and two months after radiofrequency ablation. The image of HAP was reconstructed with nMERA HRCM images at 40–70-keV, and simulated 120-kVp SCM image. A high attenuating lesion abutting IVC was noted in nMERA 40–50 keV images. This lesion was not noted in 120-kVp images taken 1 month before and after the nMERA images, which indicating the false positive. All the images were under constant window width/center (300/40) setting.

Table 3 demonstrates sensitivity, specificity, accuracy and AUC for HCC detection in the HRCM groups. Fig 5 demonstrates the receiver operating characteristic (ROC) curves of HRCM groups. The AUC of 40–50-keV images were better than 60–70-keV images. The sensitivity of 40-keV and 50-keV was higher than 60–70-keV (p < 0.05), while the specificity revealed no statistical significance (p > 0.05). All the lesions were detected in 40–50 keV nMERA images, while two false positive lesions were noted in 40-keV images. A representative case with false positive result was shown in Fig 6.

**Table 3:** Sensitivity, specificity, and AUC for detection of HCCs.

	Half reduced contrast medium			
	40-keV nMERA	50-keV nMERA	60-keV nMERA	70-keV nMERA
Sensitivity	14/14 (100%)	12/14 (85.7%)	6/14 (42.9%)	6/14 (42.9%)
Specificity	31/33 (93.9%)	33/33 (100%)	33/33 (100%)	33/33 (100%)
Accuracy	45/47 (95.7%)	45/47 (95.7%)	39/47 (82.9%)	39/47 (82.9%)
AUC (95% CI)	0.99 (0.96–0.99)	0.99 (0.98–0.99)	0.94 (0.85–0.99)	0.93 (0.83–0.99)

## Discussion

Our study results showed that compared with simulated 120-kVp linear blended images at SCM, low-energy nMERA HRCM images preserved vascular and visceral contrast enhancement and diagnostic performance, and high-energy nMERA HRCM images demonstrated adequate image quality. This finding is of practical importance because it demonstrates that multiphasic hepatic CT can be performed at a lower CM dose without compromising on diagnostic performance.

The VMI of DECT has the potential to improve image metrics such as the CNR and image noise [18, 24–26]. Hanson et al [21] compared the VMI and low-kVp images of DECT and found that low-keV VMI has more contrast enhancement and lesion conspicuity but with trade-offs for image quality and noise. They concluded that using both low- and high-keV VMI can ensure optimal lesion conspicuity and image quality in abdominal CT. Some studies have investigated the application of VMI to reduce the CM dose in abdominal CT [5, 9, 11]. Large differences in iodine concentrations between the lesion and the surrounding tissue may emphasize lesion conspicuity at lower energy levels, particularly in multiphasic hepatic CT among patients with HCC. Lv et al [5] used rapid kV switching DECT to produce VMI in abdominal CT, and they revealed that compared with 120-kVp images reconstructed using the filtered back projection algorithm, 50-keV VMI reconstructed using the iterative reconstruction algorithm enabled CM dose reduction and adequate image quality and lesion conspicuity. Although contrast enhancement was the highest at 40-keV in this study, 40-keV nMERA HRCM images also had the highest noise. Nagayama et al [9] used dual-layer DECT in multiphasic hepatic CT and found that the noise of VMI with a half-reduced CM dose was lower than that of the 120-kVp image with a full CM dose, and the noise was almost constant at different energy levels. They concluded that 40–55-keV images have equivalent or higher image quality and lesion conspicuity than 120-kVp images. These results suggest that VMI is promising for CM dose reduction. However, no study has investigated diagnostic performance in VMI. Although the introduction of nMERA in the dual-source dual-energy system has improved the CNR and conspicuity of low-contrast objects at low energy levels [18–21, 27, 28], no study has investigated the nMERA images of DECT with a reduced CM dose in abdominal CT. To the best of our knowledge, this is the first study to investigate the usefulness of nMERA images of dual-source DECT for CM dose reduction in multiphasic hepatic CT by evaluating image quality and diagnostic performance.

Our data confirmed and extended the results of previous studies [20, 27, 28] and showed that low-energy nMERA images facilitated CM dose reduction with the preservation of iodine CNR and conspicuity of the hypervascular liver tumor. The results are consistent with those obtained from other dual-energy CT technologies [5, 9, 11]. The contrast enhancement and CNR of low-keV nMERA HRCM images were not inferior to those of the simulated 120-kVp SCM images; however, nMERA HRCM images showed a substantial increase in noise at low energy levels compared with the simulated 120 kVp SCM images, which were equivalent to 70 keV nMERA HRCM images. This was reflected by the score of subjective image quality, revealing that the image quality of 40- and 50-keV nMERA HRCM images were inferior to that of the simulated 120-kVp SCM images. However, given the detrimental trade-off of image quality at low energy nMERA images, the sensitivity, accuracy and AUC for HCC detection were acceptable among 40- and 50-keV nMERA HRCM images in our study. The difference in attenuation of the HCC tumor and hepatic parenchyma was emphasized at the lower energy level in HAP. Our results suggest that when detection of hyperenhancing HCC is accounted for the clinical purpose, 40–50 keV nMERA images have the competent role for routine multiphasic hepatic CT under a reduced CM dose. However, the emphasizing of contrast attenuation at lower energy nMERA may give false positive results as shown in Fig 5, which decrease the specificity. In addition, it may be concerned whether the reduced CM dose alters the enhancing size of the tumor, and therefore affects the diagnostic performance in the small HCC.

Our study has some limitations. First, it was a retrospective study with limited numbers of patients and lesions, as this study was conducted in limited clinical settings to detect HCC recurrence post ablation. Further investigation is warranted to ascertain whether the lesion detectability of nMERA images at a reduced CM dose can be maintained when this technique is widely implemented clinically. Second, the final diagnosis is based on CT or MR within two months after RFA, and the pathological diagnosis was not obtained for all the patients. These are due to the nature of retrospective study, however, further prospective study with a larger number of patients and pathologic diagnosis is required. Although the detection of tumor recurrence is based on multiphasic hepatic CT, particularly the HAP [29–31], the restriction to only the arterial and portal venous phases is the shortcoming of the study. Another limitation is the BMI of the subjects in our study are lower (BMI < 30 kg/m^2^). Generally, the image noise increases as the body size increases [18, 20, 28]. Further study may be necessary to confirm the findings in a patient population with larger BMI.

In conclusion, nMERA images enable HRCM in multiphasic hepatic CT while preserving diagnostic accuracy. We recommend that 40–50 keV nMERA images could be applied for assessment of local tumor progression after RFA of HCC when subtle contrast enhancement differences are diagnostically important, while beware the false positive.
